# Development and Evaluation of an Immunoinformatics-Based Multi-Peptide Vaccine against *Acinetobacter baumannii* Infection

**DOI:** 10.3390/vaccines12040358

**Published:** 2024-03-27

**Authors:** Sean Jeffreys, Megan P. Tompkins, Jadelynn Aki, Sara B. Papp, James P. Chambers, M. Neal Guentzel, Chiung-Yu Hung, Jieh-Juen Yu, Bernard P. Arulanandam

**Affiliations:** 1 Department of Molecular Microbiology and Immunology, University of Texas at San Antonio, San Antonio, TX 78249, USA; sean.jeffreys@utsa.edu (S.J.); meganpt@umich.edu (M.P.T.); jadelynn.aki@utsa.edu (J.A.); james.chambers@utsa.edu (J.P.C.); m.guentzel@utsa.edu (M.N.G.); chiungyu.hung@utsa.edu (C.-Y.H.); 2 Department of Immunology, Tufts University School of Medicine, Boston, MA 02111, USA

**Keywords:** *Acinetobacter baumannii*, immunotherapeutic, vaccine, epitope vaccine, peptide vaccine, multi-drug resistant bacteria, nosocomial pathogen

## Abstract

Multi-drug-resistant (MDR) *Acinetobacter baumannii* is an opportunistic pathogen associated with hospital-acquired infections. Due to its environmental persistence, virulence, and limited treatment options, this organism causes both increased patient mortality and incurred healthcare costs. Thus, prophylactic vaccination could be ideal for intervention against MDR *Acinetobacter* infection in susceptible populations. In this study, we employed immunoinformatics to identify peptides containing both putative B- and T-cell epitopes from proteins associated with *A. baumannii* pathogenesis. A novel *Acinetobacter* Multi-Epitope Vaccine (AMEV2) was constructed using an *A. baumannii* thioredoxin A (TrxA) leading protein sequence followed by five identified peptide antigens. Antisera from *A. baumannii* infected mice demonstrated reactivity to rAMEV2, and subcutaneous immunization of mice with rAMEV2 produced high antibody titer against the construct as well as peptide components. Immunization results in increased frequency of IL-4-secreting splenocytes indicative of a Th2 response. AMEV2-immunized mice were protected against intranasal challenge with a hypervirulent strain of *A. baumannii* and demonstrated reduced bacterial burden at 48 h. In contrast, all mock vaccinated mice succumbed to infection within 3 days. Results presented here provide insight into the effectiveness of immunoinformatic-based vaccine design and its potential as an effective strategy to combat the rise of MDR pathogens.

## 1. Introduction

The prevalence of multi-drug-resistance (MDR) bacterial strains has increased yearly and has become a burden on healthcare systems globally, significantly increasing costs and mortality [1,2]. A significant contributor to this crisis is *Acinetobacter baumannii (Ab)*, a nonmotile Gram-negative bacterium. It is one of the most successful opportunistic nosocomial pathogens due to its MDR phenotype and ability to avoid desiccation while thriving in healthcare settings under selective pressure [3]. Typically, *Ab* healthcare-associated infections present as ventilator-associated pneumonia, catheter-related bloodstream/urinary infections, and wound infections in military personnel [4,5,6]. The rise in cases over recent decades results from increased usage of the intensive care unit (ICU), increased length of hospital stays, and overuse of antibacterial therapy [7,8]. Recent isolates have exhibited extreme drug resistance (XDR), such as carbapenem-resistant *Ab* (CRAB) strains, prompting the World Health Organization (WHO) to assign CRAB the highest critical priority ranking for the identification of novel drug therapies to combat this pathogen [9]. Such designations highlight the need for the development of alternative strategies to combat MDR *Ab* as current antimicrobials become less effective year after year. This emergency has prompted researchers to investigate the efficacy of immunotherapies, e.g., vaccination or monoclonal antibodies, as viable alternatives.

Many early and current immunotherapeutic developments against this pathogen have demonstrated the adaptive potential to protect against acute *Ab* infection. Studies utilizing whole-cell vaccines, such as UV-inactivated, formalin-inactivated, and live-attenuated bacteria, provide robust protection to vaccinated mice [10,11,12,13]. The robust immunogenicity and broadly protective nature of these vaccines is due to the plethora of antigens available to prime the adaptive immune response to *Ab*. Despite their protective capabilities, no preclinical evaluation of these candidates has begun. The hesitation to utilize such vaccines is mainly due to safety concerns. Thus, current *Ab* vaccine efforts are focused on developing safer protein subunit vaccines, and additional work in understanding *Ab* pathogenesis has shed light on virulence factors and novel vaccine targets. Subunit vaccines have afforded partial protection across multiple studies, showing limited capability in providing robust protection against *Ab*’s ability to adapt to selective pressures due in part to targeting a single antigenic protein in a rapidly changing pathogen [14,15,16].

With the emergence of bioinformatics, it is now possible to quickly scan entire bacterial genomes for potential vaccine candidates, a process referred to as reverse vaccinology [17]. The abundance of targets can be filtered and downselected based on sequence conservancy among different strains, antigenic prediction, solubility upon overexpression, and in silico immune system simulation. These tools accelerate vaccine target selection and design with immunoinformatic tools capable of predicting T- and B-cell epitopes of interest in each antigen [18]. These epitopes can be fused together with linkers hypothetically providing protection to multiple antigens in contrast to vaccines targeting a single antigenic protein. Fusing epitopes together is not a novel concept and has shown great success in T-cell-based vaccines due to the manner in which the immune system processes protein antigens and subsequent peptides [19]. However, in silico, predicted B-cell epitopes are linear and not conformational. Thus, it remains to be seen if such linear B-cell epitopes elicit antibodies capable of recognizing three-dimensional antigen conformations.

In the last few years, there has been a significant increase in the publication of immunoinformatic-based vaccines against various pathogens [20,21]. However, few have been assessed for their immunogenicity and protectivity in vivo. In this study, we designed and evaluated the immunogenicity and protective efficacy of an *Acinetobacter* Multi Epitope Vaccine (AMEV) consisting of predicted immunogenic peptide regions across five different *Ab* virulence factors.

## 2. Materials and Methods

### 2.1. Animals

All animal experiments were performed using 7–8-week-old C57BL/6 mice purchased from Jackson Laboratories (Bar Harbor, ME, USA). Mice were housed at the University of Texas at San Antonio in an AAALAC-accredited animal facility. All animal experiments were performed in accordance with Institutional Animal Care and Use Committee Protocol MU070.

### 2.2. Bacteria

*Acinetobacter baumannii* clinical isolate 79 (Ci79) obtained from the San Antonio Military Medical Center (SAMMC; Fort Sam Houston, San Antonio, TX, USA) was provided by Dr. James Jorgensen (University of Texas Health Science Center at San Antonio, San Antonio, TX, USA) [22,23]. Bacteria were streak-plated on Luria-Bertani (LB) agar plates supplemented with ampicillin (100 µg/mL) from a frozen stock. An overnight culture was prepared from a single colony, incubated overnight at 37 °C, subcultured the following day to an OD_600nm_ = 0.03 in fresh LB broth, and grown for 3.5 h until mid-log phase. Subcultures were centrifuged at 5000 rpm for 5 min and bacteria pellets were resuspended and washed in phosphate-buffered saline (PBS). Following the first PBS wash, bacteria were diluted to an OD_600nm_ = 0.5 (≈2 × 10^8^ CFUs/mL). This inoculum was either further diluted for the intraperitoneal (i. p.) challenge or centrifuged and concentrated for the intranasal (i. n.) challenge. Bacterial inoculum CFU/mL was determined by serial dilution and plating.

### 2.3. rAMEV2 Cloning and Purification

The rAMEV2 expression vector contained an *Ab*-leading protein sequence linked to 5 identified peptides via a rigid EAAAK amino acid linker. Peptides were joined by the short amino acid linkers, GPGPG or KK, and the resulting construct contained a 6× His tag at the C-terminus for protein purification. The *E. coli* codon-optimized nucleotide sequence for rAMEV2 was synthesized by GenScript (Piscataway, NJ, USA). The pET23a- AMEV2 vector was transformed into BL21(DE3) competent *E. coli*. rAMEV2 expression was induced in the presence of 1 mM IPTG and purified under denaturing conditions using a HisPur cobalt spin column (Thermo Fisher, Waltham, MA, USA) according to manufacturer’s guidelines. The denatured recombinant protein was refolded by serial dialysis against PBS in the presence of 2-fold diluted urea (4 to 1 M). Following final dialysis against PBS-purified rAMEV2 protein was aliquoted and stored at −80 °C until used. The purified rAMEV2 protein sequence was confirmed by standard liquid chromatography-tandem mass spectrometry (LC-MS/MS) from trypsin-digested fragments.

### 2.4. AMEV2 Vaccination and A. baumannii Challenge

C57BL/6 (7–8-weeks-old) mice were randomly divided into Mock (PBS + Adjuvant) or AMEV2 (rAMEV2 + Adjuvant) groups. rAMEV2 (10 µg/mouse) or PBS alone (100 µL/mouse) was formulated with AddaS03 adjuvant (InvivoGen, San Diego, CA, USA) at a 1:1 (*v*/*v*) ratio and injected subcutaneously on day 0. All mice were boosted with the same dose on days 14 and 28 and rested for four weeks before evaluating protective efficacy. Briefly, *Ab* Ci79 inocula containing 2 × 10^9^ CFUs/mL were prepared for intranasal challenge. Mice were anesthetized by isoflurane inhalation, and 50 µL bacteria (10^8^ CFU/mouse) was administered dropwise to the nares. Animals were monitored daily for morbidity (weight loss) and mortality for 30 days following the bacterial challenge.

### 2.5. Organ Bacterial Burden Measurement

Lungs, spleens, kidneys, and blood were collected from PBS mock, PBS + AddaS03 mock, and rAMEV2 + AddaS03 vaccinated mice (*n* = 5 per group per time point) at 24 and 48 h post intranasal challenge with *Ab* Ci79 (10^8^ CFU/mouse) in 2 mL PBS. Tissues were homogenized, serially diluted, and plated on LB agar supplemented with ampicillin (100 µg/mL) to enumerate bacterial burdens.

### 2.6. Determination of Antibody and Isotype Levels by ELISA

Microtiter plates were coated with rAMEV2 protein (500 ng/well) or peptides (1 µM per well) overnight in sodium bicarbonate buffer (pH 9.5). Plates were then washed with PBS containing 0.05% Tween 20 (PBST) and blocked with PBS containing 10% (*v*/*v*) fetal bovine serum (blocking buffer) for 1 h at room temperature (23–25 °C). Following blocking, plates were washed with PBST and sera diluted with blocking buffer were added to the plates and incubated for 2 h at room temperature. Following three washes with PBST, plates were incubated for 1 h with goat anti-mouse total Ig, IgG1, and IgG2c conjugated to HRP (Southern Biotechnology Associates, Birmingham, AL, USA) diluted 1:4000 in blocking buffer. Plates were washed three times with PBST and TMB substrate reagent (BD Biosciences, San Diego, CA, USA) was added to each well for color development. Color development was stopped after 15 min with the addition of 2 M H_2_SO_4_. Absorbance at 450 and 570 nm was recorded for each well using a microplate reader (TECAN, Männedorf, Switzerland). Endpoint titers were determined as the highest dilution with an absorbance reading of 0.1 greater than the blank absorbance reading.

### 2.7. B-Cell ELISpot Assays

The frequency of antigen-specific antibody-secreting cells was evaluated using the B-cell ELISpot assay. Spleen and bone marrow were collected 1 week after the second vaccination. PVDF membrane ELISpot plates (Millipore Sigma, Burlington, MA, USA) were coated overnight with 1 µg per well of either hen egg lysozyme (HEL, an unrelated antigen negative control), goat anti-mouse Ig (Southern Biotechnology Associates, a positive control), or rAMEV2. PBS-only wells served as a negative control. Plates were washed with PBS and blocked with RPMI-1640 supplemented with 10% (*v*/*v*) fetal bovine serum, 100 U penicillin/mL, 100 µg streptomycin/mL, and 2 mM L-glutamine (R10). 2.5 × 10^5^ Splenocytes and bone marrow cells were seeded into the wells and incubated for 6 h at 37 °C with 5% CO_2_. Following incubation, plates were washed with PBST before adding goat-anti-mouse-Ig-AP-conjugated secondary antibody (Southern Biotechnology Associates) diluted 1:5000 with 1% (*w*/*v*) bovine serum albumin (BSA) in PBS and incubated at 4 °C overnight. Plates were then washed with PBST and incubated with BCIP/NBT phosphatase substrate (SeraCare, Milford, MA, USA) for spot development. The reaction was terminated with water and dried before reading using an ImmunoSpot analyzer (CTL) as previously described [24].

### 2.8. T-Cell ELISpot Assays

T-cell reactivity was evaluated using IFNγ, IL-4, and IL-17 ELISpot assays as previously described with modification [25]. Spleens from AMEV2-vaccinated or adjuvant-only treated mock mice were isolated one-week post-second vaccination. Splenocytes were evaluated for in vitro recall with rAMEV2 (10 µg/mL) or UV-inactivated *Ab* (1 × 10^6^ CFU/well). Media-only wells served as a negative control for background, HEL (10 µg/mL) served as an antigen-specificity control, and α-CD3 (clone: 145-2C11, 1 µg/mL) served as a positive control. PVDF membrane ELISpot plates (Millipore Sigma) were coated overnight with either IFNγ (clone: AN-18, 2 µg/mL), IL-4 (clone: 11B11, 4 µg/mL), or IL-17 (clone: 17F3, 2 µg/mL) capture antibodies. The following day, plates were washed with PBS and blocked with complete R10 media while spleen tissue was collected and prepared. 5 × 10^5^ splenocytes were seeded in IFNγ and IL-4 wells, while 10^6^ cells were seeded into the IL-17 wells. Antigens at indicated concentrations were added and incubated at 37 °C with 5% CO_2_. IFNγ and IL-17 plates were stimulated for 24 h, while IL-4 plates were left for 48 h. Following the indicated incubations, plates were washed with PBS before secondary antibodies were added. Biotinylated IFNγ (clone: R4-6A2, 0.5 µg/mL), IL-4 (clone: BVD6-24G2, 2 µg/mL), and IL-17 (clone: eBioTC11-8H4, 0.125 µg/mL) detection antibodies were added to the respective wells and incubated at 4 °C overnight. Plates were then washed with PBS before incubation with Streptavidin Alkaline Phosphatase conjugate (Invitrogen, Waltham, MA, USA) for 2 h at room temperature. Following an additional wash, BCIP/NBT phosphatase substrate (SeraCare) was added for spot development. Reactions were quenched with water, followed by drying and spots were counted using an ImmunoSpot analyzer (CTL).

### 2.9. Statistical Analysis

GraphPad Prism 10.0 was used to determine statistical significance tests. Differences between mock and AMEV2 vaccinated groups were assessed using the Student’s *t* test, multiple Mann–Whitney tests, and one-way or two-way analysis of variance (ANOVA). Survival rates were analyzed with the Log-rank Mantel-Cox test. Differences were considered statistically significant when *p* ≤ 0.05.

## 3. Results

### 3.1. Immunoinformatics Based Peptide Selection

**Figure 1:**
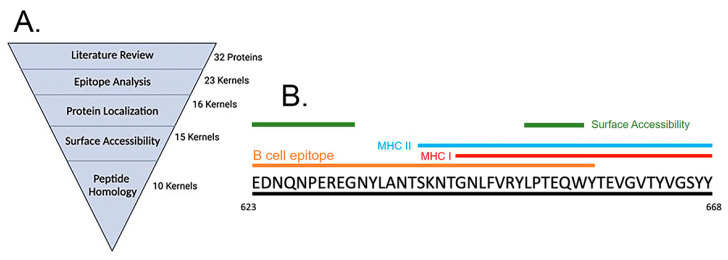
Peptide selection. (**A**) Selection of potential virulence factor targets. (**B**) Graphical representation of pTonB peptide in silico predicted regions.

The strategy for selection of T- and B-cell epitopes relevant to *Ab* pathogenesis is shown in Figure 1A. Potential vaccine candidates were selected from a literature review of antigens previously characterized for their importance as *Ab* virulence factors. A total of 32 candidate targets were analyzed with EigenBio’s proprietary epitope prediction software [26,27]. Peptides were filtered by their predicted localization in the outer membrane [28]. Additionally, each protein was analyzed for surface accessibility to ensure that the predicted B-cell epitopes were surface-exposed [29]. Lastly, peptide candidates were screened against all identified *Ab* strains for high conservancy as well as mouse and human genomes with BLASTP to limit the potential for autoimmune reaction [30]. Selected peptides satisfying the respective criteria are listed in (Table 1). A graphical representation of the in silico predicted TonB peptide is shown in Figure 1B. Additional predictions for each peptide are shown in Supplemental Figure S1.

### 3.2. Generation of an Acinetobacter Multi-Epitope Vaccine, AMEV2

**Figure 2:**
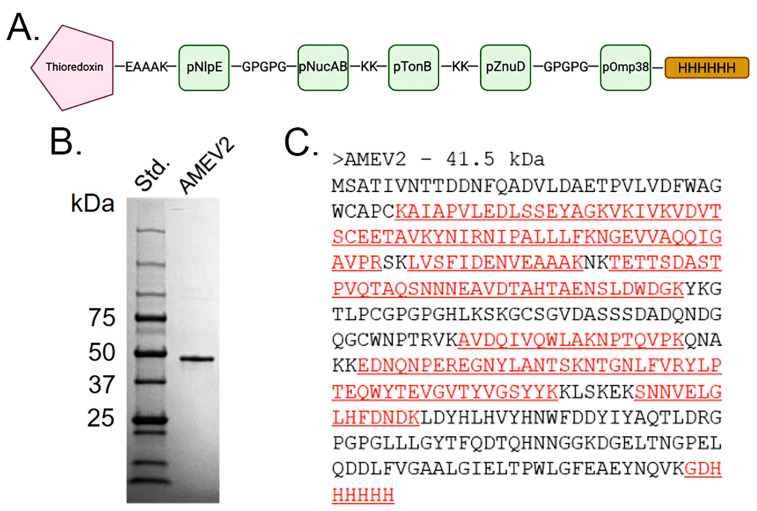
Generation of polypeptide vaccine AMEV2. (**A**) Diagram of rAMEV2 comprised of *A. baumannii* thioredoxin linked to 5 predicted immunogenic peptides and C-terminus His tag. (**B**) SDS-PAGE analysis of Coomassie-blue stained purified rAMEV2. (**C**) Amino acid sequence of rAMEV2 with matched peptides identified by LC-MS/MS in red.

The rAMEV2 construct consists of *A. baumannii* thioredoxin (TrxA) protein linked to five predicted immunogenic peptides (Table 1). Although TrxA is a well-characterized virulence factor, it also assists in improving the solubility of the rAMEV2 construct [31,32,33,34]. Peptides were joined by either GPGPG or KK linkers to limit the creation of irrelevant pseudo epitopes [35,36,37]. Each peptide plus linker was analyzed to ensure the maintenance of the B-cell epitopes. The five-peptide cluster conjugate was linked to TrxA on the N-terminus end via a rigid EAAAK linker, creating proper separation, thus reducing the possibility of interaction between the peptides and TrxA [38]. A C-terminus 6-His Tag aids in protein purification (Figure 2A). The rAMEV2 construct was cloned into the pET23a vector and transformed into BL21(DE3) *E. coli*. rAMEV2 was successfully expressed and purified to near homogeneity, as shown in Figure 2B. The identity of purified rAMEV2 was confirmed with LC-MS/MS performed by the University of Texas at San Antonio Proteomics Core (Figure 2C).

### 3.3. rAMEV2 Contains Epitopes Relevant to A. baumannii Natural Infection

**Figure 3:**
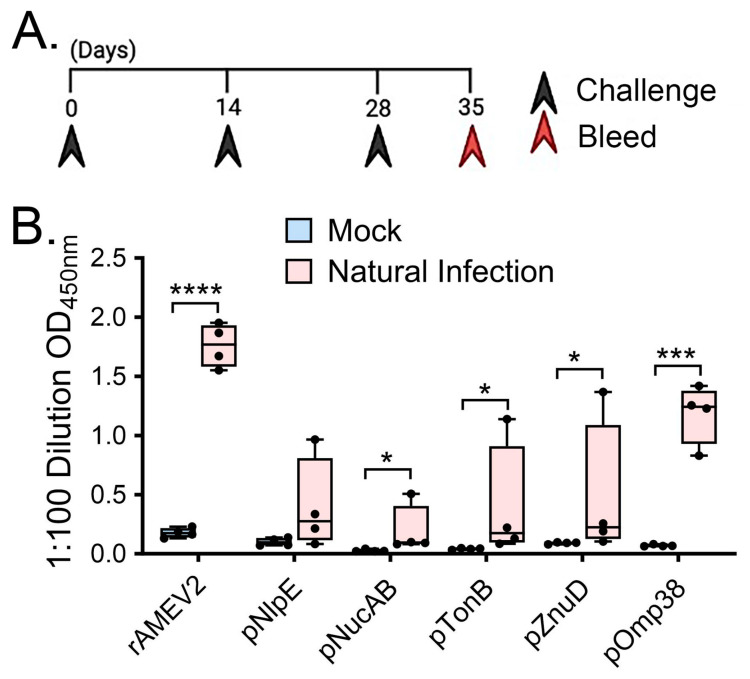
Sera from mice sensitized by sublethal *A. baumannii* infection demonstrates reactivity with rAMEV2. (**A**) Natural infection vaccination timeline for C57BL/6 mice (*n* = 4 per group). Following sublethal intraperitoneal injection of 10^5^ CFU, mice were subsequently boosted twice two weeks apart before sera were collected a week after the final boost. (**B**) Day 35 serum from *Ab*-sensitized mice diluted 1:100 was screened for reactivity against rAMEV2 and its constituent peptides by indirect ELISA (mean *±* SD). **** (*p* ≤ 0.0001; T test), *** (*p* ≤ 0.001; T test), * (*p* ≤ 0.05; Mann–Whitney) between mock and *Ab* sensitized mouse sera.

C57BL/6 mice (*n* = 4) were challenged intraperitoneally with a sublethal dose of bacteria (10^5^ CFU) and re-challenged twice 2 weeks apart subcutaneously to enhance antibody production (Figure 3A). Sera collected from these and naïve mice (*n* = 4) were screened for antibodies recognizing rAMEV2 epitopes. Compared to naïve mice, mice sensitized to *Ab* demonstrated significant reactivity to rAMEV2 (Figure 3B). The peptide components were also screened, and pOmp38 demonstrated the most significant reactivity across all mice.

### 3.4. AMEV2 Vaccination Generates Robust Humoral Immunity

**Figure 4:**
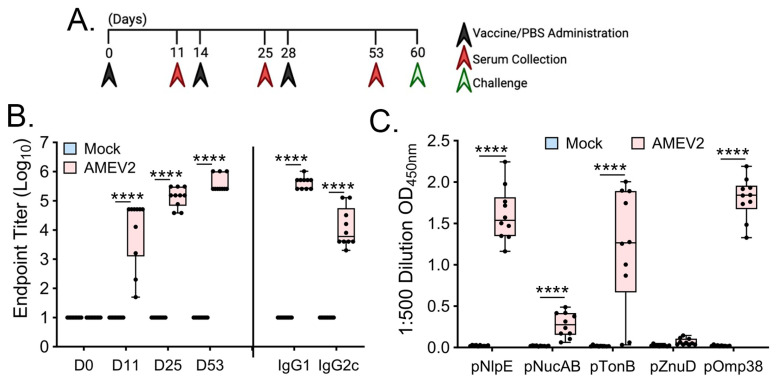
Vaccination with rAMEV2 and AddaS03 adjuvant generates a robust humoral response. (**A**) The 7–8-week-old C57BL/6 mice (*n* = 10 per group) were vaccinated subcutaneously with either PBS + AddaS03 (mock) or rAMEV2 (10 µg) + AddaS03 (AMEV2) on days 0, 14, and 28. Sera were collected prior to boosting and bacteria challenge at the indicated times. (**B**) Sera from either mock or AMEV2-vaccinated mice were serially diluted and screened against rAMEV2 for endpoint titer determination via indirect ELISA. (**C**) Day 53 sera was diluted 1:500 and checked for reactivity with rAMEV2 peptides via indirect ELISA. Data are presented as mean *±* SD. **** Significant (*p* ≤ 0.0001; T test) between mock and AMEV2 vaccinated mouse sera.

**Figure 5:**
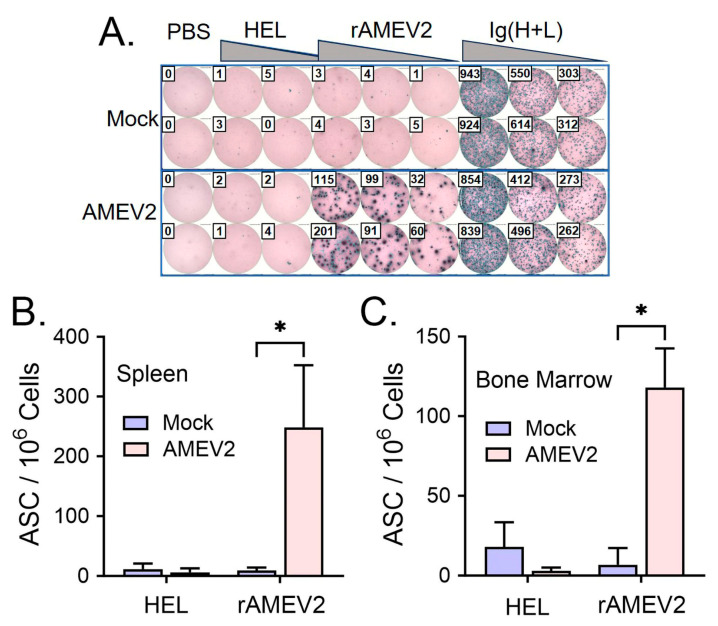
AMEV2 vaccination generates rAMEV2-specific antibody-secreting cells (ASC) in spleen and bone marrow. The 7–8-week-old C57BL/6 mice (*n* = 2 per group) were vaccinated subcutaneously with either PBS + AddaS03 (mock) or rAMEV2 (10 µg) +AddaS03 (AMEV2) on days 0 and 14. Spleens and bone marrow were recovered one week post-first boost. (**A**) Representative ELISpot readouts for B-cells that secreted antibodies reacting with PBS, hen egg lysozyme (HEL), or rAMEV2. Wells coated with goat anti-mouse Ig(H + L) served as positive control. The frequency of rAMEV2-specific ASCs was calculated in spleens (**B**) and bone marrow (**C**). Data are presented as the mean *±* SD. * Significant (*p* ≤ 0.05; T test) between mock and AMEV2 vaccinated mouse ASCs.

C57BL/6 mice (7–8-week-old, *n* = 10 per group) were vaccinated and bled according to the schedule in Figure 4A. Compared to mock sera, AMEV2 vaccinated sera shows remarkable antibody generation to the rAMEV2 construct even after a single vaccination. The rAMEV2 titer continued to increase following the two boosts (Figure 4B). Day 53 sera contained both high IgG1 and IgG2c titers to rAMEV2 (Figure 4B), with significantly more IgG1 indicative of a Th2 response [39]. Peptide ELISA demonstrated that four of five peptides contained in rAMEV2 were immunogenic with no immunodominance by a single peptide (Figure 4C). Spleens and bone marrow of vaccinated mice were investigated to determine rAMEV2-specific antibody-secreting cells one week post-first boost (Figure 5A). With no reaction to HEL, the B-cell ELISpot demonstrates specificity and high frequency of rAMEV2-specific antibody-secreting cells in spleens (Figure 5B) and bone marrow (Figure 5C) of immunized mice.

### 3.5. AMEV2 Vaccination Stimulates a Th2 Response

**Figure 6:**
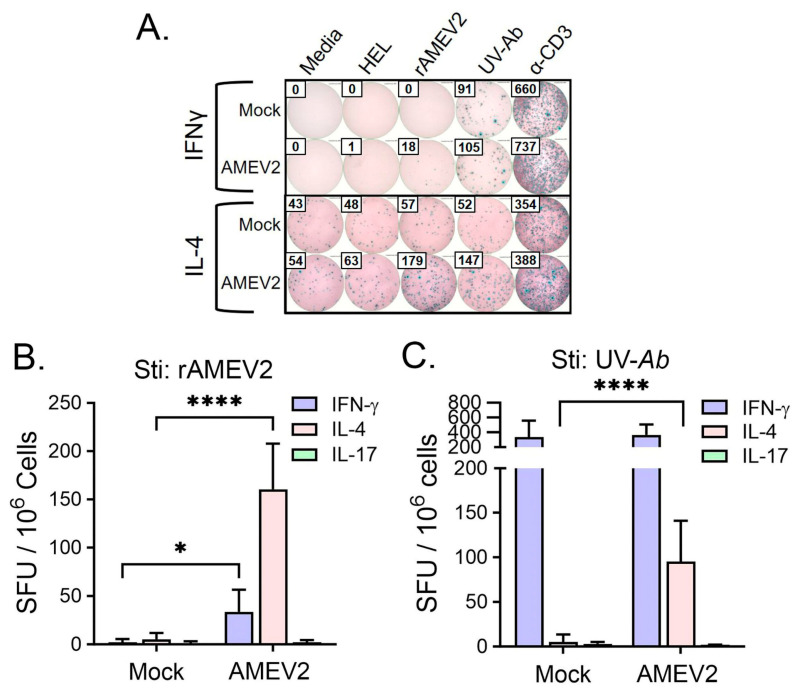
AMEV2 vaccination induces T-cell immune responses. The 7–8-week-old C57BL/6 mice (*n* = 3 per group) were vaccinated subcutaneously with either PBS + AddaS03 (mock) or rAMEV2 (10 µg) +AddaS03 (AMEV2) on days 0 and 14. Spleens were recovered one week post-first boost. (**A**) Mock or AMEV2 vaccinated splenocytes were stimulated with media, HEL, rAMEV2, UV-inactivated *A. baumannii*, and α-CD3. Detection of IFNγ, IL-4, or IL-17 spot forming units (SFU) indicated a Th1, Th2, or Th17 response, respectively. (**B**) Cytokine-secreting cells recall response to rAMEV2. (**C**) Cytokine-secreting cells recall response to UV-inactivated *Ab.* Data are presented as the mean *±* SD. **** (*p* ≤ 0.0001; T test),* (*p* ≤ 0.05; T test) between mock and AMEV2 vaccinated mouse SFUs.

We utilized the T-cell ELISpot recall assay to determine if AMEV2 vaccination increases the number of IFNγ, IL-4, or IL-17 secreting splenocytes upon restimulation with rAMEV2 or whole UV-inactivated *A. baumannii* (UV-*Ab*) compared to mock (PBS + AddaS03) vaccination. Mock splenocytes had minimal to no reactivity to HEL and rAMEV2. There was an increase in IFNγ secreting cells when exposed to UV-Ab, likely due to excess LPS from the bacteria. In contrast, AMEV2-vaccinated mice showed a significant increase in both IFNγ and IL-4, but not IL17, secreting cells upon restimulation with rAMEV2 (Figure 6B). Additionally, this bias towards a Th2 response with IL-4 secretion was observed when exposed to UV-Ab as well (Figure 6C). Background generated from LPS in the UV-*Ab* treated sample obscures the potential for a significant increase in IFNγ that was observed from rAMEV2 restimulation.

### 3.6. AMEV2 Affords Protection against Pulmonary Challenge

**Figure 7:**
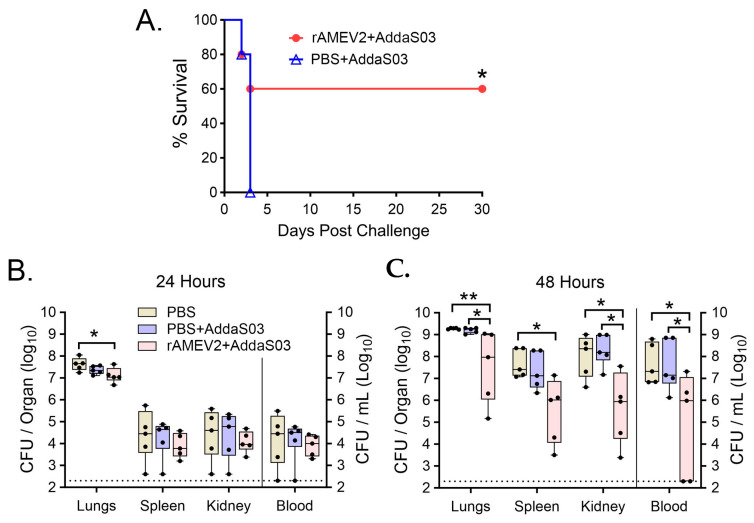
AMEV2 vaccination provides protection against pulmonary *Acinetobacter baumannii* infection. (**A**) 7–8-week-old C57BL/6 mice (*n* = 10 per group) were vaccinated subcutaneously with either PBS + AddaS03 or rAMEV2 (10 µg) + AddaS03. Mice were challenged with 50 µL (1.01 × 10^8^ CFU) *A. baumannii* strain Ci79 intranasally and monitored 30 days for survival. * (*p* ≤ 0.05; Log-rank) between mock (PBS) and AMEV2 vaccinated mouse survival status over 30 days. (**B**,**C**) 7–8-week-old C57BL/6 mice (*n* = 10 per group) were vaccinated fully with either PBS, PBS + AddaS03, or rAMEV2 (10 µg) + AddaS03 and challenged four weeks later intranasally with 1.06 × 10^8^ CFUs of Ci79. Mice were sacrificed at (**B**) 24 h and (**C**) 48 h time points, and blood and organs were harvested for determination of bacterial burden by serial dilution and plating. * (*p* ≤ 0.05; Mann–Whitney), ** (*p* ≤ 0.01; Mann–Whitney) between PBS, PBS + AddaS03, and rAMEV2 + AddaS03 vaccinated mouse bacterial burdens.

C57BL/6 mice (*n* = 10 per group) were vaccinated with rAMEV2 plus AddaS03 adjuvant or mock vaccinated with PBS plus AddaS03, as described in Figure 4A. Mice were rested for 4 weeks before being challenged intranasally with 10^8^ CFU Ci79 and evaluated for survival over 30 days. By day 3, all mock vaccinated mice had succumbed to acute infection. In contrast, 60% of AMEV2-vaccinated mice survived over 30 days (Figure 7A). To assess the protective effect of the AMEV2 vaccine on the bacterial burden, mice (*n* = 5 per group) treated with PBS alone, PBS + AddaS03, and rAMEV2 + AddaS03 were challenged intranasally. Tissues were harvested at 24 (Figure 7B) and 48 h (Figure 7C) to determine the extent of bacterial burden at the challenge site and dissemination to other target organs. At 24 h, PBS + AddaS03 and rAMEV2 + AddaS03 vaccinated mice demonstrated a slight reduction in lung bacterial burden compared to PBS mock mice, with no significant difference in bacterial dissemination. However, at 48 h, the AMEV2 vaccinated group demonstrated a significant reduction in bacterial burden in all tissues compared to PBS and adjuvant mock groups, with only the spleen showing no difference between the adjuvant and AMEV2 mice.

## 4. Discussion

Here, we describe a novel multi-peptide *Acinetobacter baumannii* vaccine designed using an immunoinformatic approach. Despite efforts over the past two decades to develop vaccines against this pathogen, none have advanced to clinical evaluation. Current efforts to develop subunit vaccines against *Ab* include whole recombinant proteins, immunogenic antigen peptides, and multi-peptides composed of short discontinuous B-cell and T-cell epitopes [40,41,42,43]. Our focus was to keep immunological sequences intact to improve T-cell-dependent antibody response, taking advantage of the chimeric nature of the construct’s potential for multivalent protection to counteract potential vaccine escape. This multivalent construct consisted of peptide segments from five virulence factors involved in *Ab* pathogenesis: NlpE, NucAB, TonB, ZnuD, and Omp38. The leader protein for this multi-peptide construct is *Ab* TrxA, an additional virulence factor well characterized by our laboratory and functions as a solubility enhancer [31,44,45]. NlpE is a copper-resistance protein involved in biofilm formation and bacterial adhesion in many MDR *Ab* strains [46]. Of the five virulence factors, NlpE is the only virulence factor that has not been evaluated previously as a vaccine candidate, although copper resistance gene knockouts have demonstrated attenuated virulence in a murine pneumonia model [47]. NucAB is an outer membrane nuclease and has been predicted in silico to be a desirable target due to its location. It is also highly conserved among *Ab* isolates [48]. Garg and coworkers have demonstrated the protective efficacy of recombinant NucAB with 20% survival following active immunization and 40% with passive immunization against pulmonary *Ab* infection. TonB is a general siderophore receptor important for iron acquisition and growth in the host [49]. A well-characterized siderophore receptor, BauA, rendered mice actively vaccinated using its recombinant form partial protection and complete protection following passive vaccination [50]. Like TonB, ZnuD is a zinc piracy receptor involved in resistance to host nutritional immunity [51]. A vaccine composed of ZnuD surface loops on a hybrid antigen afforded mice complete protection in an *Ab* bacteremia model [52]. Lastly, Omp38 belongs to the outer membrane protein A (OmpA) family, which has been thoroughly characterized as a therapeutic target against *Ab* infection [53]. It is capable of inducing apoptosis in epithelial cells and inhibits the host complement system [54,55]. Additionally, one of the first subunit vaccines against *Ab* targeted OmpA and demonstrated 50 and 90% protection following active and passive vaccination, respectively [56]. AMEV’s multivalent potential includes inhibition of biofilm formation, bacterial adhesion, nutritional immunity, and host immunomodulation. These virulence factors have shown promise as vaccine candidates individually and contain protective epitopes against *Ab* infection.

The protective epitopes within these virulence factors were determined in silico using the EigenBio epitope prediction software. Five peptides, between 40 and 60 amino acids in length, containing B- and T-cell epitopes from each virulence factor were selected. Screening each peptide against sera from mice that recovered from an *Ab* infection indicated four of the five peptides exhibited specificity for α-*Ab* antibodies. Additionally, antisera reacted strongly to the complete recombinant AMEV2 construct. In vivo, immunogenic evaluation of rAMEV2 demonstrated that four of the five predicted peptides were immunogenic and did not give rise to the formation of new junctional epitopes between the linked peptides. The Omp38 peptide demonstrated the highest reactivity to serum following *Ab* infection, indicating the likelihood of it being a protective epitope. It remains to be determined which of the five peptides is necessary for protection to optimize the AMEV construct. Overall, these in silico-selected peptides demonstrate the ability to remain immunogenic, generating effective antibodies when linked together in a multipeptide construct.

rAMEV2 subcutaneously administered with AddaS03 adjuvant generates a robust humoral response, which has been the primary focus of *Ab* vaccine development due to the acute nature of the murine infection model. This aligns with most *Ab* vaccines demonstrating the effectiveness of antibody-mediated protection with passive sera transfer and monoclonal antibody generation [11,13,14,15,56,57,58,59,60,61]. However, the impact of cell-mediated protection against *Ab* remains poorly understood, given the limited cell-mediated immunity data from whole-cell vaccines. Our laboratory has previously characterized a live-attenuated strain of thioredoxin deficient Ci79 for its cell-mediated response and observed a minor increase in IL-17 secretion but no significant elevation in IFNγ or IL-4 upon restimulation with *Ab* [60]. However, vaccination of mice with an *Ab* D-glutamate auxotrophic strain deficient in wall peptidoglycan synthesis by Cabral et al. demonstrated significant IL-4 and IL-17 responses [13]. An outer membrane vesicle vaccine demonstrated an increase in the Th2 subset (CD4^+^/IL-4^+^) of T-cells in vaccinated splenocytes, indicating the adaptive immune response to this pathogen being Th2 dominant [62]. Protein subunit vaccines, in combination with highly immunogenic adjuvants, have encouraged investigation of the relevance of cell-mediated immunity against this pathogen with various adjuvants. We have shown that rAMEV2-sensitized splenocytes are capable of secreting IFNγ and IL-4 in response to restimulation to the protein construct with significantly higher IL-4 rAMEV2 specific T-cell frequency indicative of a Th2 response. This Th2 memory response is also elicited upon restimulation with whole *Ab* bacteria. The very acute nature of these infections leaves little room for investigating a potentially effective T-cell response, but IL-4 secretion and a Th2 response could be required for modulation of the protective B-cell response. This T-cell-dependent antibody response is instrumental in enhancing B-cell activation and effective antibody production, as evidenced by AMEV2′s substantial IgG1 and IgG2c antibody titers.

Using this design, we have demonstrated partial protection by AMEV2 vaccine to an otherwise lethal intranasal dose of a virulent *Ab* strain. AMEV2 vaccination reduces bacterial burden and dissemination to other tissues, ultimately leading to abrogation of TLR4-mediated septic shock compared to mock vaccinated control mice [63,64]. The mechanisms of this seemingly antibody-mediated protection remain to be established, thus requiring further study. Partial protection afforded by the AMEV2 vaccine is in agreement with other protein subunit vaccines, but it still underperforms in comparison to complete protection achieved by whole-cell vaccination to this pathogen [10,11,12,13,58]. Discovering additional conserved protective peptides will undoubtedly expand the AMEV vaccine constructs’ effectiveness and reactivity against additional *Ab* strains. For example, in a pilot study, we observed the broad protective efficacy of the AMEV2 vaccine in a systemic challenge model against an alternative hypervirulent strain of *Ab*, AB5075. Mice (*n* = 10) fully vaccinated with rAMEV2 in conjunction with Titermax Gold adjuvant and challenged intraperitoneally achieved 80% survival over 30 days compared to the PBS mock control group, which succumbed to bacteremia after 24 h (Supplemental Figure S2). This strategy indicates that AMEV constructs can be further refined to achieve desirable vaccine candidate status as a viable immunotherapeutic alternative for combating MDR *Ab* infection.

## 5. Conclusions

With no current vaccine in clinical trials for MDR *A. baumannii,* there is a dire need for novel therapeutics to combat this pathogen. Whole-cell vaccines have demonstrated strong protection, but hesitation in their safety profile has hindered their adoption. We sought to develop a subunit protein vaccine candidate capable of eliciting multivalent protection from peptides. The AMEV vaccine candidates were developed following a review of *A. baumannii* virulence factors involved in pathogenesis. Peptides containing T- and B-cell immunogenic epitopes within these targets were identified and selected for their protein localization, surface accessibility, and high homology. The rAMEV2 construct includes five of these peptides, each from a different virulence factor, linked to an *Ab* Thioredoxin leader protein. This design resulted in a highly expressible chimeric protein. Screening rAMEV2 against mouse sera from sublethal *A. baumannii*-sensitized mice revealed antibody reactivity to epitopes contained in rAMEV2. Immunization of mice with rAMEV2 in conjunction with AddaS03 adjuvant generated a robust humoral response with high IgG1 and IgG2c titers to the construct and its constituent peptides. High frequencies of rAMEV2-specific antibody-secreting cells were measured in the spleens and bone marrow of vaccinated mice. T-cell ELISpot recall assay demonstrated a predominately Th2 mediated response in AMEV2 vaccinated splenocytes with a high frequency of IL-4 secreting T-cells upon restimulation with rAMEV2 as well as UV-inactivated Ci79 *A. baumannii*. This robust immune response afforded AMEV2 vaccinated mice 60% survival in a pulmonary infection model against hypervirulent *A. baumannii* with reduced bacterial burdens at 48 h compared to mock vaccinated mice. The immunoinformatic design of the rAMEV2 construct indicates a viable strategy for the development of novel immunotherapeutics against MDR pathogens. Ongoing studies are determining the necessity of certain peptides with the goal of continued iteration to improve the efficacy of this vaccine candidate. This multivalent peptide approach will be refined to produce a safer protein subunit vaccine that can afford protection against MDR infections in susceptible populations.

## Figures and Tables

**Table 1:** Predicted immunogenic peptide segments selected for construction of AMEV2.

Peptide Antigen	Derived from Protein Name	NCBI Reference Sequence	Peptide Range	Peptide Size	Peptide Amino Acid Sequence
pNlpE	Copper resistance protein NlpE	WP_000749178.1	23–68	46	NKTETTSDASTPVQTAQSNNNEAVDTAHTAENSLDWDGKYKGTLPC
pNucAB	ExeM/NucH family extracellular endonuclease	WP_000847239.1	604–656	53	HLKSKGCSGVDASSSDADQNDGQGCWNPTRVKAVDQIVQWLAKNPTQVPKQNA
pOmp38	OmpA family protein	WP_000777885.1	29–85	57	LLLGYTFQDTQHNNGGKDGELTNGPELQDDLFVGAALGIELTPWLGFEAEYNQVKGD
pTonB	TonB-dependent siderophore receptor	WP_001998816.1	623–668	46	EDNQNPEREGNYLANTSKNTGNLFVRYLPTEQWYTEVGVTYVGSYY
pZnuD	Zinc piracy TonB-dependent receptor ZnuD	WP_000899872.1	497–538	42	LSKEKSNNVELGLHFDNDKLDYHLHVYHNWFDDYIYAQTLDR

## Data Availability

Data are contained within the article and Supplementary Materials.

## Supplementary Materials

The following supporting information can be downloaded at https://www.mdpi.com/article/10.3390/vaccines12040358/s1, Figure S1: Graphical representation of in silico prediction of AMEV2 peptides; Figure S2: AMEV2 vaccination provides protection against systemic *Acinetobacter baumannii* infection.
