# Supplementary material for: Development and Evaluation of an Immunoinformatics-Based Multi-Peptide Vaccine against Acinetobacter baumannii Infection
Source: Vaccines (Basel). 2024 Mar 27;12(4):358. doi: 10.3390/vaccines12040358 (PMC11054912; doi:10.3390/vaccines12040358)
Supplement: Supplementary file 1 [file vaccines-12-00358-s001.zip › vaccines-2914713-supplementary.pdf]

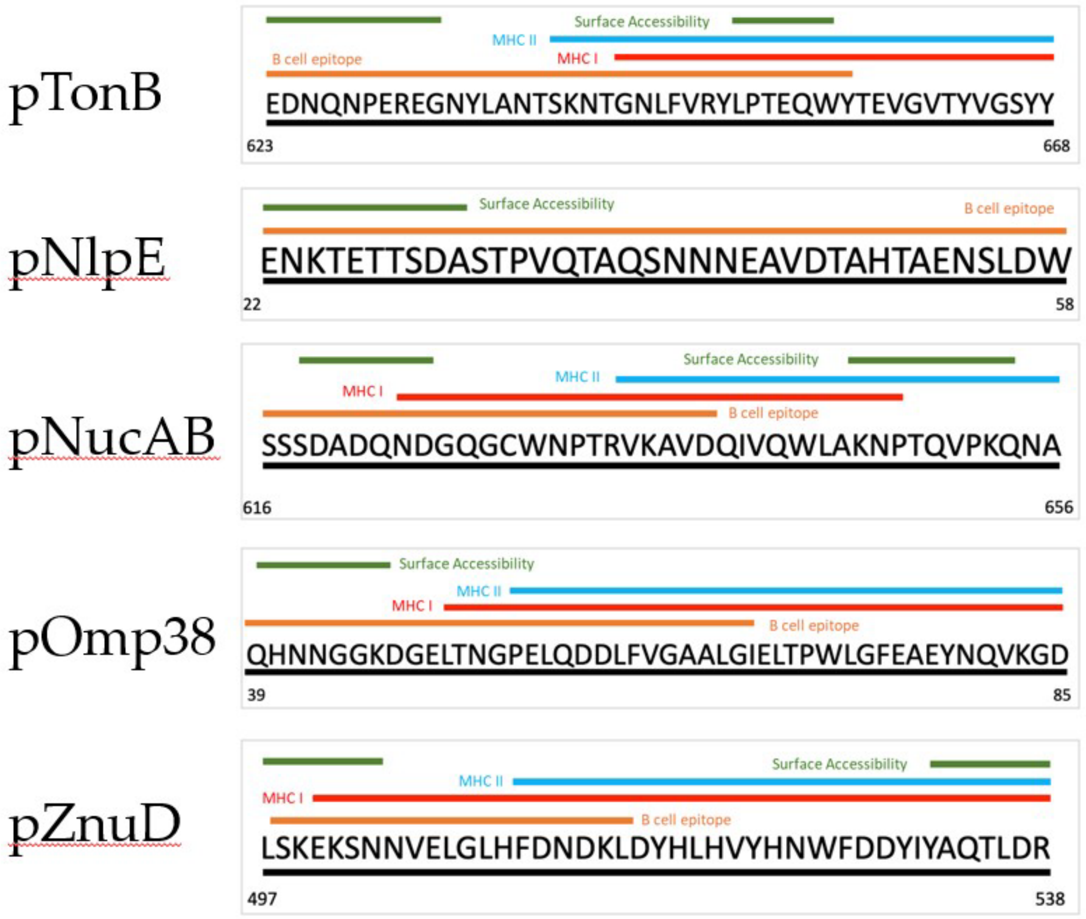


Figure S1. Graphical representation of *in silico* prediction of rAMEV2 peptides with EigenBio epitope prediction software.

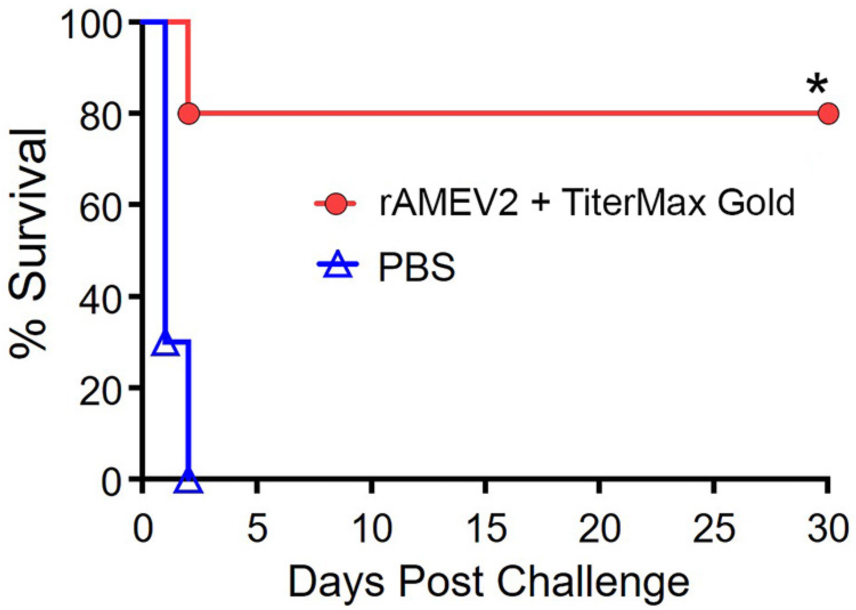


Figure S2. AMEV2 vaccination provides protection against systemic *Acinetobacter baumannii* infection. (A) 7-8 week old C57BL/6 mice (n = 10 per group) were vaccinated subcutaneously with either PBS or rAMEV2 (5 µg) + TiterMax Gold adjuvant. Mice were challenged with 100 µL (2 x $10^6$ CFU) (4 x $LD_{50}$) *A. baumannii* strain AB5075 intraperitoneally and monitored 30 days for survival. * $p < 0.05$ (Log-rank) between PBS and AMEV2 vaccinated mouse survival status over 30 days.
